# Changes in expression of nuclear factor kappa B subunits in the ovine thymus during early pregnancy

**DOI:** 10.1038/s41598-022-21632-3

**Published:** 2022-10-21

**Authors:** Ling Yang, Chunjiang Cai, Shengya Fang, Shaopeng Hao, Taipeng Zhang, Leying Zhang

**Affiliations:** School of Life Sciences and Food Engineering, Hebei University of Engineering, Handan, 056038 China

**Keywords:** Immunology, Zoology

## Abstract

There is a pregnant maternal immunological tolerance that protects the fetus and promotes its growth, and nuclear factor kappa B (NF-κB) family participates in the regulation of innate immune and adaptive immune responses. The thymus is related to establishing central tolerance, and early pregnancy has effects on expression of a good number of genes and proteins in the maternal thymus in sheep. However, it is unclear whether early pregnancy changes expression of NF-κB subunits in the ovine thymus. In this study, the thymic samples were collected from day 16 of non-pregnant ewes, and days 13, 16 and 25 of pregnant ewes, and the expression of NF-κB members (NF-κB1, NF-κB2, RelA, RelB and c-Rel) was analyzed through real-time quantitative PCR, Western blot and immunohistochemical analysis. The results showed that *c-Rel* mRNA and protein upregulated at day 25 of pregnancy, and *NF-κB1* mRNA and proteins increased at days 16 and 25 of pregnancy, and *RelB* mRNA and proteins enhanced during early pregnancy. However, expression levels of NF-κB2 and RelA were decreased during early pregnancy, but upregulated from day 13 to 25 of pregnancy. In addition, the RelA protein was located in the epithelial reticular cells, capillaries and thymic corpuscles. This paper reported for the first time that early pregnancy induced expression of NF-κB1, RelB and c-Rel, but inhibited expression of NF-κB2 and RelA in the maternal thymus during early pregnancy, which is involved in the central immune tolerance, and helpful for successful pregnancy in sheep.

## Introduction

Placenta and decidua form key immunological barriers to sustain maternal tolerance, and protect the fetus, promote its growth during pregnancy in placental mammals. However, there are immunological similarities and differences at the maternal–fetal interface between human and mouse pregnancies^1^. Ruminant pregnancy creates unique challenges for immune systems, which are caused by the immune interactions between the fetus and uterine endometrium, as well as high concentration of circulating progesterone (P4) and conceptus signals^2^. The endocrine status causes alterations in both inter- and intracellular signaling molecules to modulate the immune system during early pregnancy in cattle, and has effects on subsequent fertility^3^. Conceptus signaling (interferon-tau, IFNT) modulates the maternal innate immune system to avoid conceptus rejection through both paracrine and endocrine manners during early pregnancy in ruminants^4,5^. It has been reported that IFNT has effects on immune tissues, including bone marrow^6^, thymus^7^, spleen^8,9^ and lymph node^10,11^ during the early stages of pregnancy in sheep.

The thymus plays key roles for the development of T lymphocytes, which contributes to establishing central tolerance, and exporting naïve T cells to the periphery in humans^12^. The thymus can produce double negative (DN) T-cells that increase significantly in pregnant women. DN T-cells contribute to a Th2 bias at maternal–fetal interface, which are necessary for the maintenance of pregnancy^13^. Successful pregnancy outcome is implicated in the regulation of maternal regulatory T cells at the maternal–fetal interface, and regulatory T cells are partly derived from the thymus, which improve maternal tolerance to the fetus in pregnancy^14^. It has been reported that early pregnancy affects expression of P4 receptor, P4-induced blocking factor, T-helper cytokines, prostaglandin synthases, melatonin receptors 1, cluster of differentiation 4 (CD4), gonadotropin releasing hormone and its receptor in the maternal thymus in sheep^7,15–18^. In addition, toll-like receptor pathway and complement system are involved in the immune regulation of the maternal thymus during early pregnancy in sheep^19,20^. Therefore, the ovine maternal thymus indeed undergos significantly changes during pregnancy.

Nuclear factor kappa B (NF-κB) family consists of five members (NF-κB1, NF-κB2, RelA, RelB and c-Rel) that are involved in regulating the innate immune and adaptive immune responses^21^. NF-κB signaling contributes to immune regulation and tolerance induction in the thymus that plays essential roles in central tolerance induction by negative selection of T cells^22^. Expression of RelB subunit of NF-κB is required for the development of thymic medulla and dendritic cells in mice^23^. NF-κB and type I interferon signalings contribute to the final maturation of thymocytes into naive T cells in the thymus, and T cells acquire competence to proliferate and emigrate^24^. NF-κB is involved in the regulation of innate and adaptive immune responses during pregnancy, but dysregulation of NF-κB leads to the premature termination of pregnancy^25^. Circulating maternal P4 remains high level throughout pregnancy in humans, and P4 receptor directly interacts with NF-κB to maintain myometrial quiescence during pregnancy^26^. It is suggested that early pregnancy may change the expression of NF-κB subunits in the thymus. Therefore, the objective of this study was to explore the expression of NF-κB1, NF-κB2, RelA, RelB and c-Rel in the maternal thymus during early pregnancy in sheep.

## Results

### Relative expression levels of NF-κB1, NF-κB2, RelA, RelB and c-Rel mRNA in the thymus

**Figure 1:**
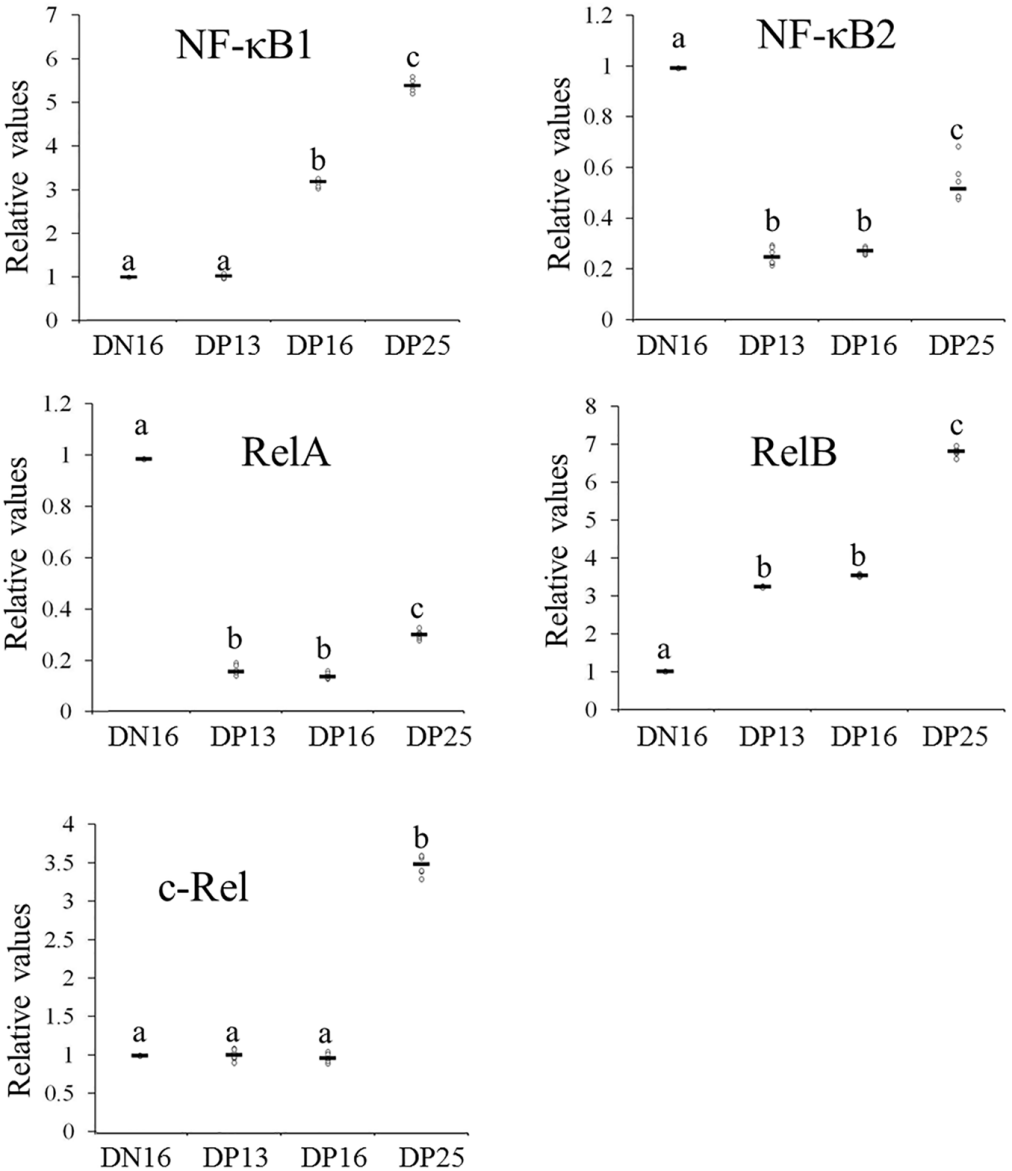
Relative expression values of *NF-κB1*, *NF-κB2 RelA*, *RelB*, and *c-Rel* mRNA in the thymus from non-pregnant and pregnant ewes (n = 6 for each group). Note: DN16 = day 16 of the estrous cycle; DP13 = day 13 of pregnancy; DP16 = day 16 of pregnancy; DP25 = day 25 of pregnancy. Significance is denoted by letters; if two groups have different letters, this indicates that the difference between these groups was significant with *P* < 0.05.

For the expression of *NF-κB1*, a significant increase in expression was seen at day 16 of pregnancy compared to day of 13 pregnancy, and a further increase from these levels was seen at day of 25 pregnancy (*P* < 0.05; Fig. 1). Expression levels of *RelB* at days 13 and 16 pregnancy were almost same, and there was an increase from day 16 to 25 of pregnancy. Furthermore, expression level of *c-Rel* was upregulated only at day 25 pregnancy (*P* < 0.05). However, the relative expression levels of *NF-κB2* and *RelA* were downregulated during early pregnancy, but there was an increase from day 16 to 25 of pregnancy (*P* < 0.05; Fig. 1).

### Expression of NF-κB1, NF-κB2, RelA, RelB and c-Rel proteins in the thymus

**Figure 2:**
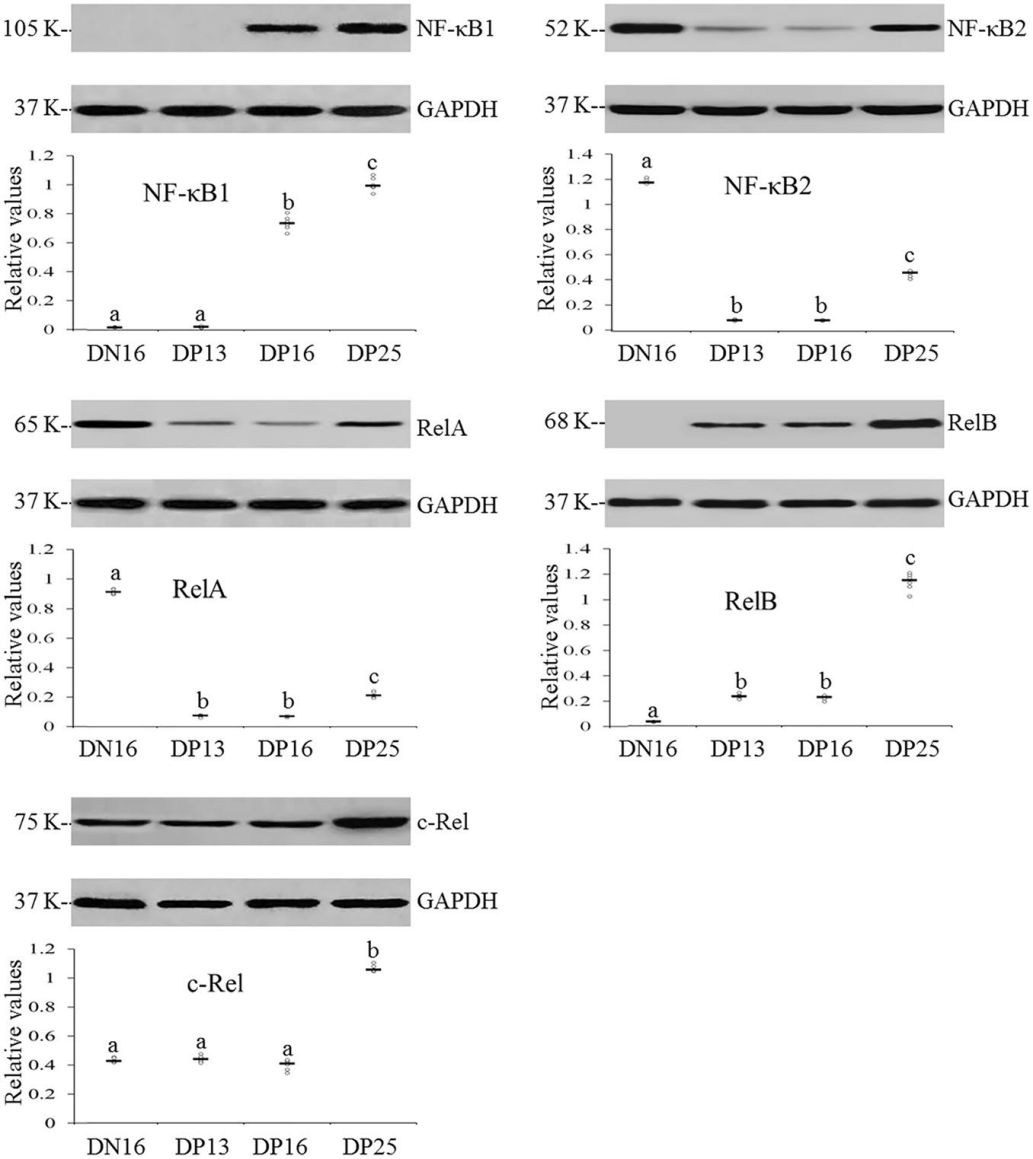
Expression of NF-κB1, NF-κB2 RelA, RelB, and c-Rel proteins in the thymus from non-pregnant and pregnant ewes (n = 6 for each group). Note: DN16 = day 16 of the estrous cycle; DP13 = day 13 of pregnancy; DP16 = day 16 of pregnancy; DP25 = day 25 of pregnancy. Significance is denoted by letters; if two groups have different letters, this indicates that the difference between these groups was significant with *P* < 0.05.

It was showed in the Fig. 2 that NF-κB1 protein was undetected at day 13 of pregnancy, and on day 16 of the estrous cycle, but the expression level of NF-κB1 protein was increased at days 16 and 25 of pregnancy (*P* < 0.05). RelB protein was undetected on day 16 of the estrous cycle, but upregulated on day 25 of pregnancy (*P* < 0.05). The protein level of c-Rel on day 25 of pregnancy was higher than on day 16 of the estrous cycle, and on days 13 and 16 of pregnancy (*P* < 0.05), but there was no significant difference among day 16 of the estrous cycle, and on days 13 and 16 of pregnancy (*P* > 0.05; Fig. 2). Nevertheless, early pregnancy induced downregulation of NF-κB2 and RelA proteins, but NF-κB2 and RelA proteins increased on day 25 of pregnancy (*P* < 0.05; Fig. 2).

### Immunohistochemistry for RelA protein in the thymus

**Figure 3:**
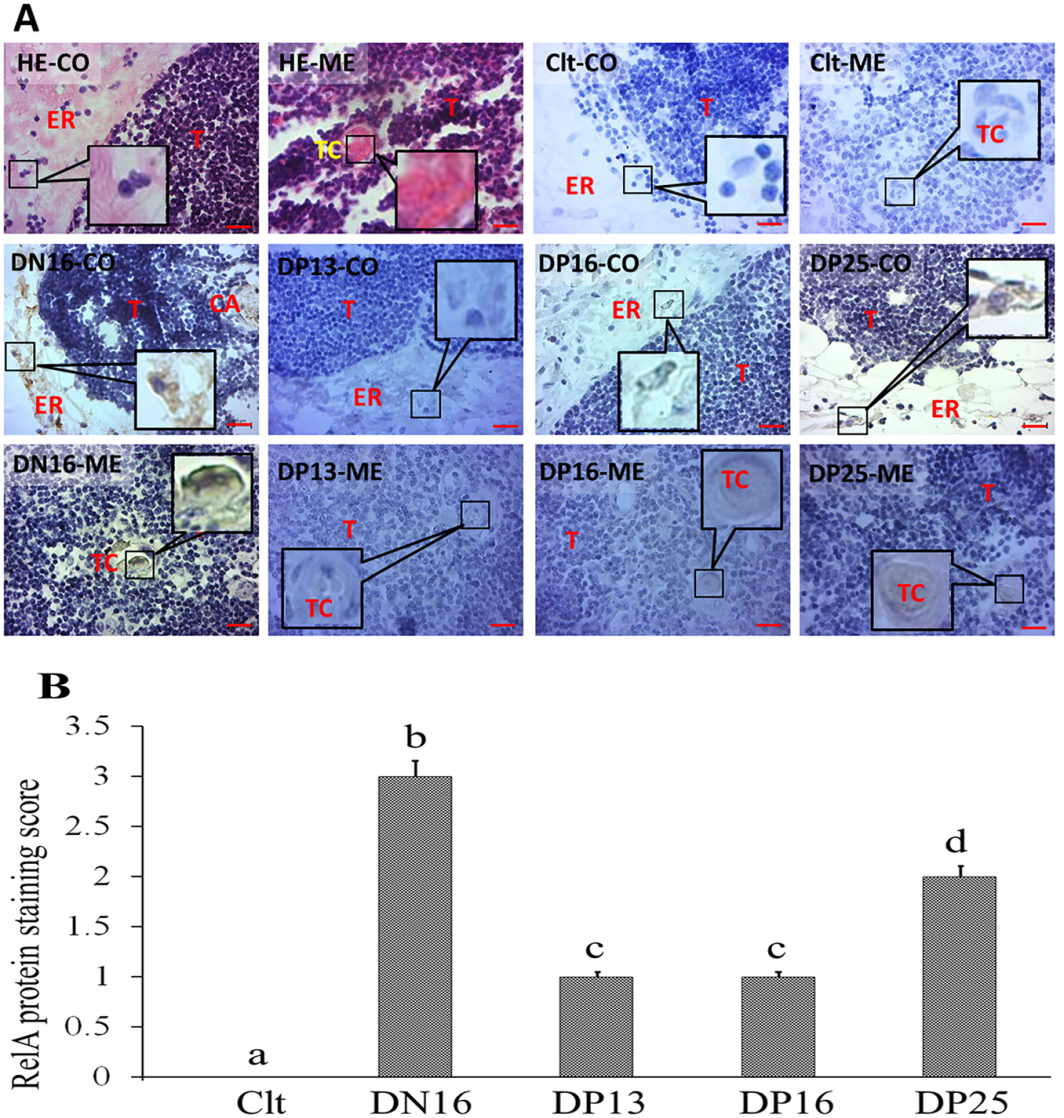
Localization of RelA protein in the thymus. (**A**) Representative immunohistochemical localization of RelA protein in the thymus from non-pregnant and pregnant ewes (n = 6 for each group). The thymus is divided into the cortex (CO) and the medulla (ME). Note: HE = stained by haematoxylin and eosin; Ctl = negative control; DN16 = day 16 of the estrous cycle; DP13 = day 13 of pregnancy; DP16 = day 16 of pregnancy; DP25 = day 25 of pregnancy; T = thymocyte; ER = epithelial reticular cell; CA = capillary; TC = thymic corpuscle. Bar = 20 µm. (**B**) Staining score for RelA protein. Significance is denoted by letters; if two groups have different letters, this indicates that the difference between these groups was significant with *P* < 0.05.

The immunohistochemistry for the RelA protein was limited to the stromal cells, capillaries and thymic corpuscles (Fig. 3). The staining intensities for RelA protein were 0, 3 + , 1 + , 1 + , and 2 + for the negative control, the thymuses from day 16 of the estrous cycle, and thymuses from days 13, 16, and 25 of pregnancy, respectively (Fig. 3). The staining intensity was as follows: 0 = negative; 1 +  = weak; 2 +  = strong; 3 +  = stronger.

## Discussion

In this study, the levels of *NF-κB1* mRNA and protein were increased at days 16 and 25 of pregnancy. As a transcription factor, NF-κB1 participates in the development and survival of lymphocytes and lymphoid organs to control immune responses in the immune system^27^. NF-κB1 protein is expressed in the epithelial compartment of the endometrium, which is involved in the implantation process at the time of implantation in humans^28^. NF-κB1 deletion results in the decrease in the size of endometrial implants^29^. NF-κB1 protein is expressed in the uterus at day 4.5 post-coitus, suggesting that transcriptions of some candidate genes are activated or suppressed by NF-κB1 around the implantation period^30^. Therefore, the upregulation of NF-κB1 in the maternal thymus may be related to the embryo implantation during early pregnancy period.

Our data indicated that early pregnancy suppressed the expression of NF-κB2, but NF-κB2 upregulated at day 25 of pregnancy. NF-κB2 is related to placental corticotropin-releasing hormone production at both transcriptional and translational levels, which controls the length of human gestation^31^. NF-κB2 positively regulates pro-labor genes in the human placenta through signal transducer and activator of transcription 3^32^. NF-κB2 is implicated in the increase of maternal plasma corticotropin-releasing hormone abundance with the pregnancy advances, which regulates the length of human gestation^33^. NF-κB2 regulates the development of medullary thymic epithelial cells that are necessary for the establishment of central self-tolerance in mice^34^. Therefore, the upregulation of NF-κB2 at day 25 of pregnancy may contribute to the establishment of self-tolerance in ewes.

Our results revealed that mRNA and protein of RelA downregulated in the thymus during early pregnancy, but upregulated at day 25 of pregnancy. In addition, the RelA protein was located in the epithelial reticular cells, capillaries and thymic corpuscles. The endometrial luminal and glandular epithelial cells increase expression of RelA in women with recurrent implantation failure, which is adverse for endometrial receptivity in humans^35^. The RelA is downregulated in the T-cells from pregnant women, which results in suppression of Th1 cytokine production, is beneficial for pregnancy success^36^. There is a negative interaction between RelA and P4 receptor, which is related with maintenance of pregnancy and immunosuppression^37^. It has been reported that P4 receptor is upregulated in maternal thymus during early pregnancy in sheep^7^, which is related with the upregulation of serum P4 level. Nevertheless, the upregulation of RelA in the trophoblastic cells contributes to the trophoblast invasiveness during pregnancy^38^. There is a thymic involution during pregnancy, which is related with the changed levels of steroids and hormones through an endocrine manner to meet the immunological prevention of foetal rejection^39^. Thus, this finding supports the idea that the decrease of RelA may be due to the high level of serum P4, and the upregulation of RelA at day 25 of pregnancy may be favorable for embryo implantation.

Our results demonstrated that early pregnancy induced upregulation of RelB, and RelB further increased from day 16 to 25 of pregnancy. There is a strong constitutive RelB activation in decidual endothelial cells, which can avoid pregnancy failure^40^. Deleterious mutations in RelB result in patients with combined immunodeficiency and autoimmunity, and RelB absence alters T cell maturation in the thymus to cause the autoimmune features in humans^41^. NF-κB1-RelB dimers contribute to regulatory T cells activation^42^, and regulatory T cells from the thymus improve maternal tolerance to the fetus in pregnancy^14^. RelB plays an essential role in the medullary thymic epithelial cell development, which control T-cell tolerance^43^, but disruption of RelB is related with multiorgan inflammation and hematopoietic abnormalities in mice^44^. Therefore, the upregulation of RelB and NF-κB1 in the maternal thymus may be associated with regulatory T cells activation, which is helpful for normal pregnancy.

In this study, c-Rel increased at day 25 of pregnancy. There is a strong expression of c-Rel in extravillous trophoblast, which contributes to the invasion, migration of choriocarcinoma cell lines^45^. NF-κB transcription factor c-Rel is essential for the development of thymic Foxp3^+^ CD4 regulatory T cell, and also important for peripheral homeostatic proliferation of regulatory T cells^46^. NF-κB subunit c-Rel is required for medullary thymic epithelial cells differentiation, and controls the development of mature medullary thymic epithelial cells to ensure central immune tolerance^47^. Thus, the upregulation of c-Rel at day 25 of gestation may contribute to the central immune tolerance, and be beneficial for embryo implantation during early pregnancy in sheep.

In conclusion, there was an upregulation of NF-κB1, RelB and c-Rel, but the expression levels of NF-κB2 and RelA were downregulated during early pregnancy. Furthermore, there were increases in expression levels of NF-κB2 and RelA from day 16 to 25 of pregnancy, and the RelA protein was limited to the epithelial reticular cells, capillaries and thymic corpuscles. Therefore, it is suggested that early pregnancy has effects on the maternal thymus to change the expression of NF-κB family, which may contribute to the central immune tolerance, and be beneficial for successful pregnancy in sheep.

## Methods

### Animals and experimental design

Multiparous Small-tail Han ewes (17 to 19 months of age) were housed at a farm in China, and fed a diet meeting nutrient recommendations of the NRC (National Research Council, 2007). The ewes were randomly allotted into four groups (n = 6 for each group), as pregnant females on days 13, 16 and 25, and estrous cyclic females on day 16. There were observations for estrous behavior three times daily in the presence of caudaepididyectomized rams, and the day of estrus was considered to be day 0. The ewes of the estrous cyclic group were not mated, but the ewes of the other three groups were allowed to mate (day 0) two times, 12 h apart, with rams of proven fertility. The ewes were slaughtered on day 16 of the estrous cycle, and day 13, 16, and 25 of gestation, and thymuses were sampled from all ewes at necropsy. Pregnancy was confirmed based on the presence of embryonic trophoblast in the uterus. Thymic sections (0.5 cm^3^) were fixed in fresh 4% paraformaldehyde in phosphate buffered solution (PBS, pH 7.4), and the remaining portions were snap-frozen in liquid nitrogen until RNA or protein extraction.

### RNA extraction and RT-qPCR assay

Total RNA from the frozen thymic tissue was extracted using TRNzol reagent (Tiangen Biotech Co., Ltd., Beijing, China) according to manufacturer’s instruction. Quantity and purity of the total RNA were assessed using the NanoDrop Lite spectrophotometer (Thermo Fisher Scientific, Wilmington, DE, USA) through measuring the absorbance ratio of all samples at 230 and 260 nm, and 260/230 values ranged from 2.0 to 2.2. After the possible genomic DNA removal, 1 μg of total RNA was used for cDNA synthesis according to manufacturer’s recommendations (FastQuant RT kit, Tiangen Biotech). A Bio-rad CFX96 real-time PCR system (Bio-Rad Laboratories, Hercules, CA, USA) was used to perform qPCR with a SuperReal PreMix Plus kit (Tiangen Biotech). The primer sequences of *NF-κB1*, *NF-κB2*, *RelA*, *RelB*, *c-Rel* and *GAPDH* were designed and synthesized by Shanghai Sangon Biotech Co., Ltd. (Table 1), and the primer product was sequenced for checking specificity. The qPCR procedures included 95 °C for 10 s, 60–62 °C (60 °C for *NF-κB1* and *NF-κB2*, 61 °C for *c-Rel*, 62 °C for *RelA* and *RelB*) for 20 s, and 72 °C for 25 s, and the number of PCR cycles was 40. A housekeeping gene (glyceraldehyde-3phosphate dehydrogenase, *GAPDH*) was used for normalization and fold change calculations, and the cycle threshold (Ct) was used for calculating fold changes in relative abundance of mRNA transcript. Mean Ct values from the mean of estrous cyclic group were used as reference points for the fold-change calculation according to the 2^-ΔΔCt^ analysis method^48^.

**Table 1:** Primers used for RT-qPCR.

Gene	Primer	Sequence	Size (bp)	Accession numbers
*NF-κB1*	Forward	CAAGCACAAGAAGGCAGCACAAC	113	XM_027970852.2
	Reverse	CAGCCATCAGCAGCAGCAGAC		
*NF-κB2*	Forward	GCCTGCTGAATGCCCTGTCTG	146	XM_042238744.1
	Reverse	CTCTGTTTCCTGTTCCACCGACTG		
*RelA*	Forward	TGGCGAGAGGAGCACAGACAC	92	XM_027959295.2
	Reverse	TGACCAGGGAGATGCGGACTG		
*RelB*	Forward	CGCTGACCTCTCCTCGCTCTC	93	XM_015100238.3
	Reverse	AAGCCGAAGCCATTCTCCTTGATG		
*c-Rel*	Forward	TCCTCCTCTGCGTCCATCTCAAG	104	XM_004005929.4
	Reverse	GTGGGGTGGGCGATTGATGAC		
*GAPDH*	Forward	GGGTCATCATCTCTGCACCT	176	NM_001190390.1
	Reverse	GGTCATAAGTCCCTCCACGA		

### Western blot analysis

The total proteins from thymic samples were extracted as described previously^19^. The protein samples (10 μg/lane) were separated on 12% SDS-PAGE gels, and then transferred to polyvinylidene fluoride membranes (Millipore, Bedford, MA, USA). Blot analysis was performed with a mouse anti-NF-κB1 monoclonal antibody (Santa Cruz Biotechnology, Inc., sc-8414, 1:1000), a mouse anti-NF-κB2 monoclonal antibody (Santa Cruz Biotechnology, sc-7386, 1:1000), a mouse anti-RelA monoclonal antibody (Santa Cruz Biotechnology, sc-8008, 1:1000), a mouse anti-RelB monoclonal antibody (Santa Cruz Biotechnology, sc-166416, 1:1000), and a mouse anti-c-Rel monoclonal antibody (Santa Cruz Biotechnology, sc-6955, 1:1000) at 4 °C overnight, respectively. Then, the membranes were washed and incubated with goat anti-mouse IgG-HRP (horseradish peroxidase) secondary antibody (Biosharp, BL001A, 1:2000) for 1 h at room temperature. Chemiluminescent substrate was used according to the manufacturer’s instructions (Tiangen Biotech) to detect immunoreactive protein. An anti-GAPDH antibody (Santa Cruz Biotechnology, sc-20357, 1:1000) was applied as an internal control protein. The blots were exposed to X-ray film, and densitometry of autoradiograms was performed using Quantity One V452 (Bio-Rad Laboratories). Relative expression levels of target proteins were normalized using the GAPDH.

### Immunohistochemical analysis

Paraffin sections (5 μm) were used for immunohistochemical localization of RelA protein as described previously^19^. Citrate buffer (0.01 M, pH 6.0) was used for antigen retrieval, and endogenous peroxidase activity was removed by fixing sections in 3% hydrogen peroxide in methanol. The sections were blocked in 5% goat serum for 1 h at room temperature, and then incubated with the mouse anti-RelA monoclonal antibody (Santa Cruz Biotechnology, sc-8008, 1:200). The sections were further incubated with the secondary antibody (Biosharp, BL001A, 1:2000) for 45 min at room temperature. For the negative control, antiserum-specific isotype was used instead of the anti-RelA antibody at the same protein concentration. Immunohistochemical localization of RelA in the thymus was visualized using a DAB kit (Tiangen Biotech) according to the manufacturer’s instructions. Digital images were captured using a light microscope (Nikon Eclipse E800, Tokyo, Japan) with digital camera DP12. The semi-quantitative analysis for the tissue slides were performed through the images independently by 4 observers, and the immunostaining intensities of the different thymic samples from different ewes (n = 6 for each group) were rated in a blinded fashion. RelA staining histological subtypes were evaluated by assigning an immunoreactive intensity of a scale of 0 to 3, as described previously^49^. Some sections were stained by haematoxylin and eosin (HE).

### Statistical analysis

The data that reflected fold changes were analyzed using least-squares ANOVA with the general linear models procedures of the Statistical Analysis System Package version 9.1 for Windows (SAS Institute, Cary, NC, USA). The qPCR and Western blot data were presented as the mean (± standard error of the mean), and the Tukey's multiple-comparison test was used for multiple comparisons between each group. Experimental groups consisted of six replicates. Data were considered statistically significant when *P* values were less than 0.05.

### Ethics approval and consent to participate

All experimental procedures were performed in accordance with the Guide for Care and Use of Agriculture Animals in Research and Teaching, and were approved by the Hebei University of Engineering Animal Care and Use Committee (HUEAE 2019-017). The study was carried out in compliance with the ARRIVE guidelines, and humane animal care and handling procedures were followed throughout the experiment. All methods were carried out in accordance with relevant guidelines and regulations.

## Supplementary Information

Supplementary Information 1.

Supplementary Information 2.

Supplementary Information 3.

Supplementary Information 4.

Supplementary Information 5.

## Data Availability

All data generated or analyzed during this study are included in this published article and its additional files.
