# Supplementary material for: Changes in expression of nuclear factor kappa B subunits in the ovine thymus during early pregnancy
Source: Sci Rep. 2022 Oct 21;12:17683. doi: 10.1038/s41598-022-21632-3 (PMC9587240; doi:10.1038/s41598-022-21632-3)
Supplement: Supplementary file 1 — Supplementary Information 1. [file 41598_2022_21632_MOESM1_ESM.pdf]

Additional file 1 Relative expression values of mRNA

Item	DN16	DP13	DP16	DP25
NF-κB1	1	1.02	3.23	5.46
NF-κB2	1	0.25	0.27	0.52
RelA	1	0.15	0.14	0.30
RelB	1	3.24	3.55	6.81
c-Rel	1	1.01	0.96	3.48
