# Supplementary figures and images for: Changes in expression of nuclear factor kappa B subunits in the ovine thymus during early pregnancy
Source: Sci Rep. 2022 Oct 21;12:17683. doi: 10.1038/s41598-022-21632-3 (PMC9587240; doi:10.1038/s41598-022-21632-3)

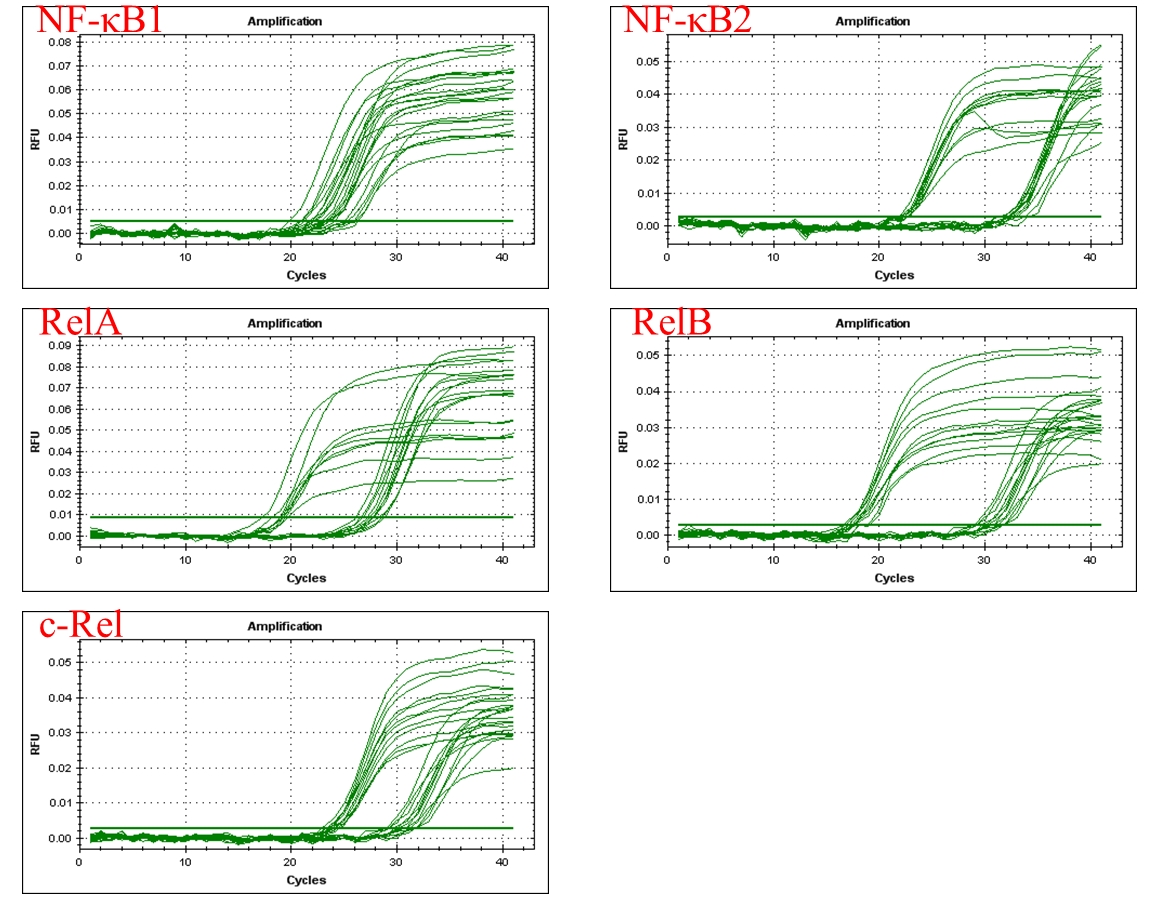

Supplement: Supplementary file 2 — Supplementary Information 2. [file 41598_2022_21632_MOESM2_ESM.jpg]

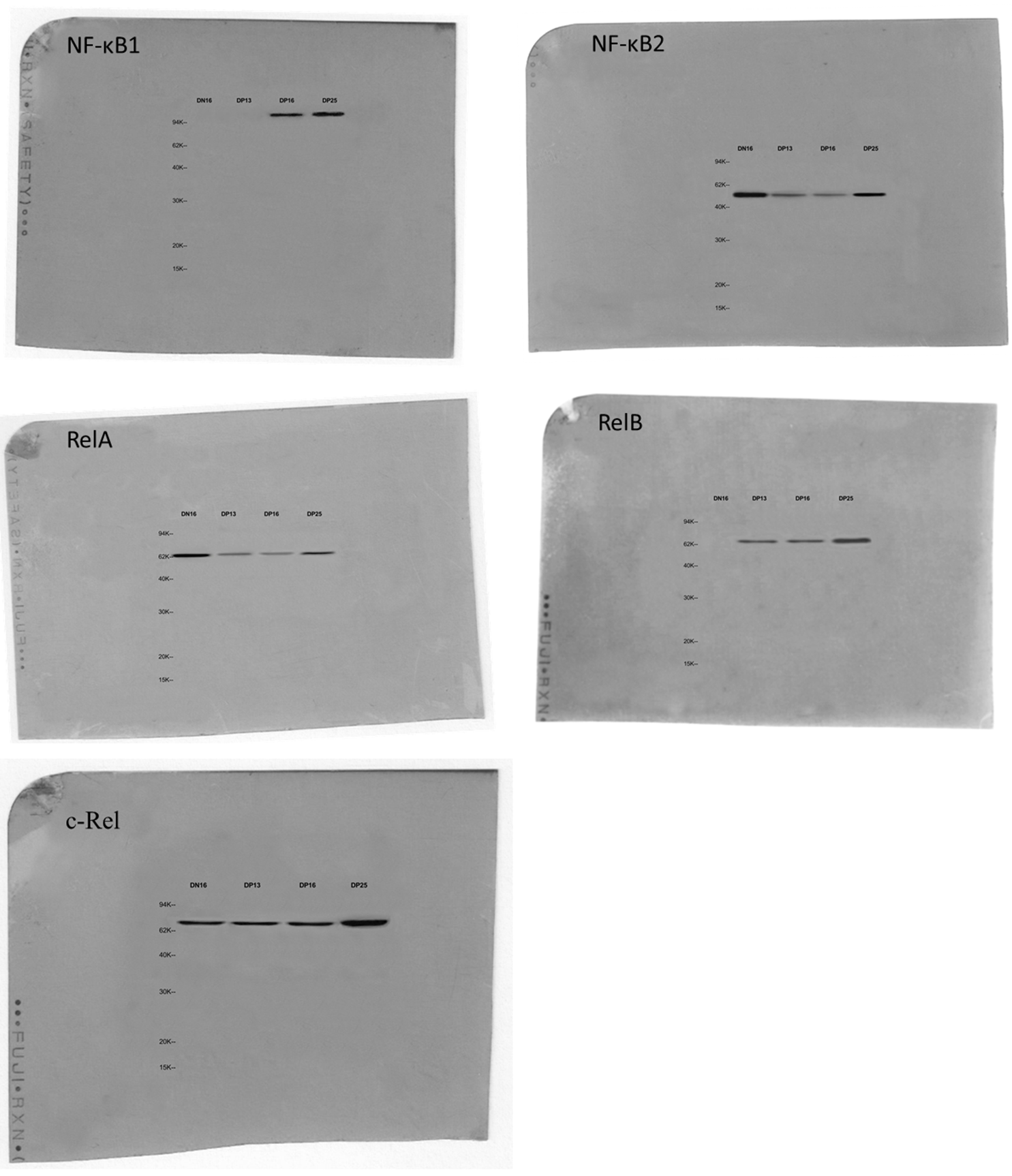

Supplement: Supplementary file 4 — Supplementary Information 4. [file 41598_2022_21632_MOESM4_ESM.tif]

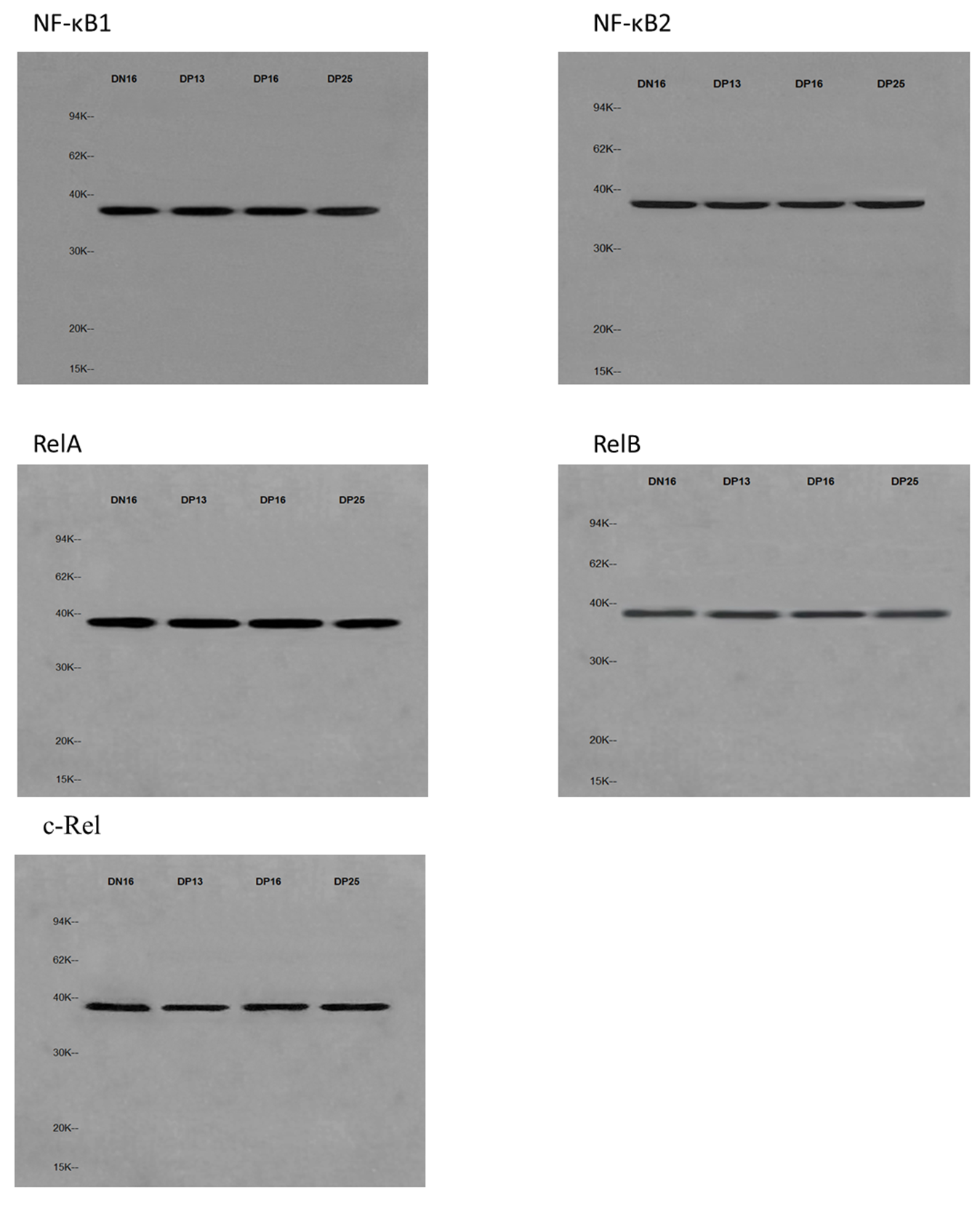

Supplement: Supplementary file 5 — Supplementary Information 5. [file 41598_2022_21632_MOESM5_ESM.tif]
