# Supplementary material for: Changes in expression of nuclear factor kappa B subunits in the ovine thymus during early pregnancy
Source: Sci Rep. 2022 Oct 21;12:17683. doi: 10.1038/s41598-022-21632-3 (PMC9587240; doi:10.1038/s41598-022-21632-3)
Supplement: Supplementary file 3 — Supplementary Information 3. [file 41598_2022_21632_MOESM3_ESM.pdf]

Additional file 3 Relative expression values of proteins

| Item | DN16 | DP13 | DP16 | DP25 |
|---|---|---|---|---|
| NF-κB1 | 0.018 | 0.021 | 0.727 | 0.981 |
| NF-κB2 | 1.181 | 0.087 | 0.085 | 0.462 |
| RelA | 0.925 | 0.075 | 0.071 | 0.221 |
| RelB | 0.032 | 0.232 | 0.226 | 1.132 |
| c-Rel | 0.412 | 0.421 | 0.396 | 1.028 |
